# Magnetically Active Cardiac Patches as an Untethered, Non‐Blood Contacting Ventricular Assist Device

**DOI:** 10.1002/advs.202000726

**Published:** 2020-11-27

**Authors:** Hongri Gu, Thibaud Bertrand, Quentin Boehler, Christophe Chautems, Nikolay V. Vasilyev, Bradley J. Nelson

**Affiliations:** ^1^ Institute of Robotics and Intelligent Systems ETH Zurich Zurich CH‐8092 Switzerland; ^2^ Department of Cardiac Surgery Boston Children's Hospital Harvard Medical School Boston MA 02115 USA

**Keywords:** cardiac patches, magnetic actuation systems, magnetically active cardiac patches, untethered devices, ventricular assist devices

## Abstract

Patients suffering from heart failure often require circulatory support using ventricular assist devices (VADs). However, most existing VADs provide nonpulsatile flow, involve direct contact between the blood flow and the device's lumen and moving components, and require a driveline to connect to an external power source. These design features often lead to complications such as gastrointestinal bleeding, device thrombosis, and driveline infections. Here, a concept of magnetically active cardiac patches (MACPs) that can potentially function as non‐blood contacting, untethered pulsatile VADs inside a magnetic actuationsystem is reported. The MACPs, which are composed of permanent magnets and 3D‐printed patches, are attached to the epicardial surfaces, thus avoiding direct contact with the blood flow. They provide powerful actuation assisting native heart pumping inside a magnetic actuation system. In ex vivo experiments on a healthy pig's heart, it is shown that the ventricular ejection fractions are as high as 37% in the left ventricle and 63% in the right ventricle. Non‐blood contacting, untethered VADs can eliminate the risk of serious complications associated with existing devices, and provide an alternative solution for myocardial training and therapy for patients with heart failure.

## Graphical abstract

Cardiac patches become multifunctional platforms on epicardial surfaces for advanced therapy. By integrating permanent magnets on the cardiac patches, magnetically active cardiac patches can provide powerful actuations that assist heart pumping inside a magnetic actuation system. These new cardiac patches have potential to function as non‐blood contacting, untethered ventricular assist devices for patients with heart failure.

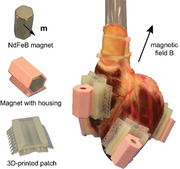

Cardiac patches are engineered materials and structures which are attached to epicardial surfaces to repair heart muscle damage caused by myocardial infarction.^[^ ^1^, ^2^, ^3^ ^]^ The patches, which contain biomaterials and living cells, could deliver stem cells to the damaged area of the heart muscles. These stem cells will eventually differentiate into cardiomyocytes and replace the scar tissue to recover the heart function. Compared to conventional injections of stem cells into the blood stream, cardiac patches show a significant improvement in delivery efficiency and functionalities.^[^ ^2^ ^]^ Recently, researchers integrated new functions into the cardiac patches, including a microneedle array that can penetrate deep into the scar tissue,^[^ ^4^ ^]^ flexible electronics for disease monitoring,^[^ ^5^ ^]^ and a reservoir that can continuously deliver drugs from outside the body.^[^ ^6^ ^]^ These studies show the potential to turn cardiac patches into multifunctional platforms on the epicardial surfaces for advanced therapy and disease management.^[^ ^7^, ^8^, ^9^, ^10^, ^11^ ^]^

To date, most cardiac patches do not provide significant mechanical actuation to the heart, making them unsuitable for treating end‐stage heart failure (HF). HF occurs when the heart is unable to pump sufficient blood to meet the body's needs. It affects 26 million people worldwide and is the leading cause of death in our society.^[^ ^12^ ^]^ Patients suffering from heart failure often require mechanical circulatory support provided by ventricular assist devices (VADs) to assist cardiac circulation. Most of the current VADs are used to bypass the diseased ventricle of the heart by rerouting blood through an artificial lumen in a mechanical pump.^[^ ^13^, ^14^, ^15^ ^]^ Clinically used VADs are durable pumps with portable battery systems, which allow weeks of support for patients with end‐stage HF, including in ambulatory settings.^[^ ^14^ ^]^However, as the blood flow comes in contact with the foreign surfaces of the pump, users are required to routinely administer anticoagulate medications.^[^ ^16^, ^17^, ^18^ ^]^ In addition, current pumps provide a nonpulsatile flow, which leads to a loss in high‐molecular‐weight multimer of von Willebrand factor, and gastrointestinal bleeding in up to 30% of patients.^[^ ^19^ ^]^ A new type of VAD is currently under development, which directly compresses the heart using soft actuators or pressurized cups that either cover the entire epicardial surface or target just one of the diseased ventricles.^[^ ^10^, ^20^, ^21^, ^22^, ^23^, ^24^, ^25^ ^]^

Both types of VADs require a cable connected to an external power source to allow the VADs to work continuously. This cable, known as a driveline, also requires an entry point into the human body and is the most common site of VAD‐related infections.^[^ ^26^ ^]^ The shearing and torsion of the driveline against the tissue can disrupt the adherent interface, allowing organisms to enter and grow along the driveline.^[^ ^27^, ^28^ ^]^ VAD‐related infections occur in 18–59% of patients after implantation.^[^ ^26^ ^]^ This affects the outcome of the mechanical circulatory support to a significant degree, including both early and long‐term mortality. A VAD that uses wireless energy transmission has been developed recently.^[^ ^29^ ^]^ However, the wireless energy transfer efficiency is limited to the coupling between the electromagnetic coils, which is sensitive to the relative coil positions.^[^ ^30^ ^]^

Here, we present magnetically active cardiac patches (MACPs) that are surgically implanted on the surface of the heart and can be remotely actuated through externally generated magnetic fields. The patient is positioned inside the magnetic field throughout operation of the device. This device provides a unique solution enabling the use of cardiac patches with VAD functions. The MACPs have the following features:

1) They are completely untethered and prevent driveline‐related infections. The MACP will only function inside the magnetic actuation system (MAS).
2) They can augment native pulsatile contractions of the ventricles thus providing physiologic circulatory support and avoiding complications associated with gastrointestinal bleeding.
3) They are non‐blood contacting. By avoiding direct contact between foreign surfaces and the blood flow, such a device design eliminates potential thrombosis formation and other complications. Patients would not require anticoagulation medications.
4) Their design and actuation can be customized for individual patients.^[^ ^31^ ^]^ By tuning the magnetization direction and size of the patches, we can achieve complex actuation patterns for the left and right ventricles, to match the natural motions of individual patient's heart muscles.

The system consists of two parts: an external MAS and MACPs implanted on the epicardial surfaces, as shown in **Figure** **1**. The MAS generates preprogrammed dynamic magnetic fields in the working environment and applies torques directly to the MACPs to achieve mechanical compression of the heart. Please note, the MACPs will only function when the patient is positioned inside the MAS (Figure 1a). In this study, the MAS is an eight‐coil electromagnetic navigation system used to generate the magnetic field.^[^ ^32^, ^33^ ^]^ We performed proof‐of‐concept *ex vivo* experiments using a fresh pig's heart obtained on the day of the experiment.

**Figure 1:**
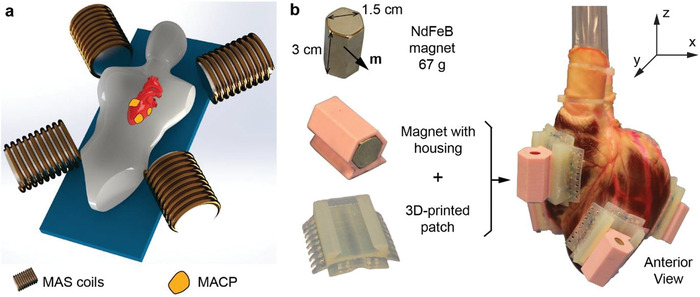
Concept and MACP design. a) Schematic illustration of functioning MACPs inside an MAS. The MAS could have different designs with several electromagnets fixed around the working environment, four coils are shown in this illustration. In the experiment, an eight‐coil MAS was used. b) Left: Structure of the MACP design; each MACP is composed of a NdFeB magnet, a magnet housing, and a 3D‐printed patch to fix on the epicardial surface. Right: Four MACPs are attached to the epicardial surfaces. Two patches are placed on the left ventricle and two on the right ventricle. The patches are placed at a suitable distance apart to prevent magnetic dipole–dipole interactions between neighboring magnets.

The structures of the MACPs are shown in Figure 1b. There are three components in one MACP: a 3D‐printed semiflexible patch, a 3D‐printed rigid magnet housing, and a permanent magnet. We chose a hexagon NdFeB magnet (length: 3 cm, hexagon length: 0.87 cm) with the magnetization being perpendicular to the long axis. The magnet has a dipole moment of *m* = *vM* = 5.44 A m^2^, where *v* is the volume of the NdFeB magnet and *M* is the magnetization of the material. The magnetic torque *τ* generated on the MACP in an external magnetic field *B* can be expressed as *τ* = *m* × *B*. For example, the maximum possible torque generated in the 80 mT magnetic field is 0.435 N m. The 3D‐printed patch has a curved surface, corresponding to the epicardial surface. The fins on the 3D‐printed patch allow us to suture the patch onto the heart using standard surgical sutures for attachment. We designed separate magnet housings to easily swap the magnetization direction relative to the heart during the experiments. The NdFeB magnet used in this study weights 67 g. The 3D‐printed magnet housing and flexible patches are 6.5 and 9.5 g, respectively. The total weight of each MACP is 83 g.

We prepared the heart using the following procedure: first, the heart was cleaned with warm water to remove all the thrombi, as any thrombi remaining in the ventricles would take up the volume of the ventricle, making the measurement inaccurate. The left and right atria were removed, and both tricuspid and mitral valves were sealed with special caps, as shown in Figure S1B (Supporting Information). The removal of the atria prevented the blood from flowing back to the atrium during the ventricular contraction, which gave us a better estimation of the performance. Two polycarbonate tubes (inner diameter: 1.2 cm) were inserted past the pulmonary and aortic valves, and were sealed with zip ties and cyanate glue. By doing this, both left and right ventricles were sealed, and the tubes were connected externally. We then filled both ventricles with colored water to make sure that there was no leakage. After sealing the heart, we sutured four MACPs onto the epicardial surface (two on the left ventricle and two on the right ventricle) using standard surgical sutures to ensure sufficient bonding between the cardiac patches and the epicardial surfaces. Sutures were placed in the areas of the epicardium away from the major coronary arteries. The two pairs of patches were positioned strategically opposing each other, in order to achieve large volume displacement from the respective ventricle (Figure 1b, right). For the left ventricle, the anterior patch was positioned on the apical area, and the posterior patch was positioned on the mid‐ventricular area at the projection of the mid‐portion of the posterior papillary muscle. For the right ventricle, the anterior patch was positioned on the outflow tract 1 cm below the projection of the pulmonary valve, and the posterior patch was positioned on the inflow at the projection of the base of the anterior papillary muscle. The magnets were then manually assembled onto the 3D‐printed patches. The detailed procedures can be found in the Supporting Information. The total preparation time was about 3 h, and during this time the heart was massaged and moisturized to maintain tissue elasticity. Before applying the magnetic field to the MACPs, we measured the initial relaxed volume of both ventricles by filling them with water and subtracting the amount of liquid remaining in the tube. The relaxed volumes inside the left and right ventricles were 21.4 and 26.2 mL, respectively.

We tested the static performances of the MACPs with a maximum magnetic field strength of 80 mT, and the dynamic performances with 60 mT. Considering the limitations of the dynamic response of the MAS itself, we only tested the alternating magnetic fields at 0.25 Hz. We studied four different magnetic arrangements as shown in **Figure** **2**a–d. In this figure, panels (a) and (b) have net dipole moments, while panels (c) and (d) have zero dipole moments within each arrangement. The net dipole moment is the sum of the dipole moments from all four magnets inside the MACPs. In arrangements (a) and (b), we observed that the heart rotated during the actuations, as shown in Figures S2 and S3 (Supporting Information), while no obvious rotations were detected in (c) and (d) (Figure 2) configurations. We think this is due to the net dipole in the magnet arrangements, as this dipole determines the rotation direction. The heart rotated inside the *x*–*z* plane in Figure S2 (Supporting Information), and *y*–*z* plane in Figure S3 (Supporting Information), respectively. This rotation is clearly undesirable; therefore, the zero net dipoles are the preferred arrangements (Figure 2c,d).

**Figure 2:**
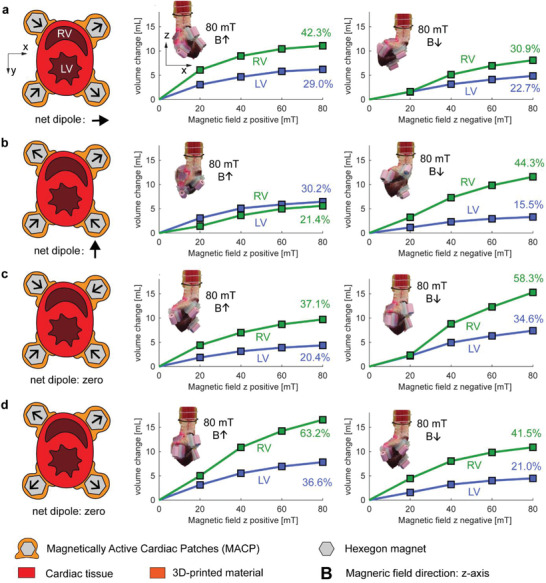
Static performance of various magnet arrangements. a–d) Schematic of four magnetic arrangements are shown in the short‐axis view. A black arrow on each hexagonal magnet shows the dipole directions. The graphs show the static pumping performances with various magnetic field strengths and with the magnetic field vector aligned on the *z*‐axis in positive and negative directions. The full step response with respect to time can be found in Figure S6 (Supporting Information). The insets show the heart positions for a magnetic field of 80 mT on the *z*‐axis with positive and negative directions.

In the static experiments, the pumping volume did not increase linearly with the magnetic field strength. This is due to the nonlinearity of the magnetic torque (*τ* = *m* × *B*) and the stiffening of the heart muscle under mechanical stretching. We calculated the ejection fraction by dividing the volumetric change by the relaxed volume in the ventricles. In the zero net dipole arrangements, maximum ejection fractions of 36.67% and 63.2% were reached for the left and right ventricles, respectively. As a comparison, the ejection fraction of a healthy human heart is about 55–70%. An ejection fraction lower than 25% is one of the indications for VAD implantation.^[^ ^34^ ^]^ We also observed a better pumping performance in the right ventricle in most cases. This can be explained by the anatomical differences between the left and right ventricles; the left ventricle has thicker muscle walls than the right ventricle, making compressions more difficult when using external actuators. Further improvements could be achieved by changing the magnet size for the MACPs to match the pumping volume to suit the individual patient's needs.

When comparing **Figures** 2 and **3**, we can see a correlation between static and dynamic performances. The magnetic configurations that show good static pumping performances also show good dynamic pumping performances (Video S1, Supporting Information). Due to the large parameter spaces, we only selected four magnet configurations to test the performances. The results indicate the promising effects of using magnetic fields to directly actuate MACPs inside the patient. Optimization and customization of the system could be achieved by varying the magnetization direction, magnet sizes, and the magnetic field oscillation patterns in the system.

**Figure 3:**
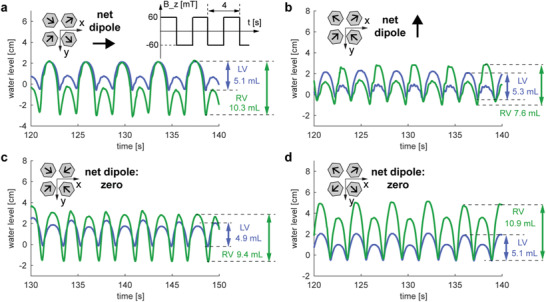
Dynamic performances under oscillating magnetic fields from −60 to 60 mT in *z*‐direction. a–d) The magnet arrangements correspond to Figure 2a–d. Under an oscillating magnetic field in the *z*‐direction (from −60 to 60 mT), the water levels in both tubes connected to the left and right ventricles were recorded to represent the pumping volumes of the MACPs. Four magnetic configurations (identical to Figure 2) were tested and the maximum volumetric changes are marked.

We have shown experimentally that MACPs can potentially function as VADs. This concept provides an untethered solution as an alternative to existing VAD devices, fundamentally allowing pulsatile augmentation of native ventricle contractions, avoiding direct blood contact with the device components, and eliminating driveline‐related infections. However, there are a few limitations to the current system. First, MACPs currently require a bulky MAS to function, which limits the application scenarios. We believe that this system could be useful in two situations: 1) as a temporary support for in‐hospital patients who are immobilized, e.g., in an intensive care unit, and 2) as a “training” support aimed at myocardial regeneration in patients who have HF with reduced ejection fraction and who are unresponsive to medical therapy, but with the option to reverse remodeling of the myocardium. Currently, mechanical circulatory support is not part of the standard care for the second group of patients, who usually receive cardiac resynchronization therapy (CRT). However, 30% of CRT recipients do not show any beneficial responses.^[^ ^35^ ^]^ Muscle regeneration using external magnetic actuation has so far only been demonstrated in rodent models of skeletal muscle ischemia.^[^ ^36^ ^]^ Ferrogel was implanted into an injured skeletal muscle that was subsequently noninvasively stimulated for 5 min every 12 h using an external permanent magnet. After 2 weeks, this stimulation resulted in enhanced muscle regeneration and an approximately threefold increase in the maximum contractile force of the treated muscle.^[^ ^36^ ^]^ There are substantial differences between myocardial and skeletal muscles, and chronic animal studies for our approach will be required.

Since the MAS used in this study was not designed for this specific application, higher magnetic fields and faster responses could be achieved by designing an MAS dedicated to this purpose. A higher magnetic field would provide stronger actuations. Importantly, this would also reduce the magnet size inside the MACPs. Improved dynamic responses in an MAS could cover the range of natural beating frequencies of the human heart, providing better synchronization with the systolic contraction phase.

The second limitation is the attachment procedures of MACPs onto the epicardial surface. In this study, we directly sutured the MACPs onto the heart muscle; however, strong hydrogel tissue adhesives are currently being developed,^[^ ^37^, ^38^, ^39^ ^]^ which would lead to less invasive and faster attachments in the near future.

Future works should focus on the development of a portable MAS for VAD applications. Using this specific system, more experiments, including in vivo experiments on the porcine model, would be possible. We can use the real‐time heart beat signals from the surface electrogram to synchronize with the native ventricular actuation patterns.^[^ ^40^ ^]^ Phase differences could be explored to optimize the volumetric output of the heart. With improved field strength, the weight and volume of the MACP could be reduced, and the development of advanced tissue adhesives would eliminate the need for manual sutures during the experiments.

## Experimental Section

##### 3D‐Printed Patches and Magnets

The patch and the sealing cap were printed by Form 2 (FormLabs). The durable resin (FLDUCL02, FormLabs) was chosen because the material is semirigid with an excellent mechanical resilience to large deformations. All printed parts were cured inside the FormCure (FormLabs) for 1 h at 60 °C. The Young's modulus was about 31.8 MPa after curing, in accordance with the manual. The housing of the magnet was printed by a fused deposition modeling (FDM) 3D‐printer using polylactic acid (PLA) filaments. The thickness of the housing surrounding the magnet was 1 mm. The hexagon prism NdFeB magnet was purchased from HKCM with N35 grade, with a hexagon length of 8.7 mm and a height of 30 mm. The NdFeB magnet used in this study weighed 67 g. The 3D‐printed magnet housing and flexible patches were 6.5 and 9.5 g, respectively. The total weight of each MACP was 83 g. The flux density inside the magnet was 1.17 Tesla.

##### Heart Preparation

A fresh pig's heart was purchased from the abattoir on the morning of the experiment. The pericardium was removed first, and then the aorta and the pulmonary artery were cut a few centimeters away from their ventricular base. The heart was then rinsed with warm running water in order to remove the remaining blood and thrombi that could reduce the actual volume of the ventricles. The atria were sealed using stereolithography (SLA) 3D‐printed “caps,” which were inserted at the top of the atrioventricular valves and sutured onto the muscle tissue of the heart once the atrial tissue had been removed. Polyvinyl chloride (PVC) tubes (inner diameter: 12 mm, outer diameter: 16 mm) were inserted into the aorta and pulmonary artery up to the aortic and pulmonary valves to allow the water to be discharged from the ventricles during the recreated systole. Finally, the patches were sutured onto the ventricular walls following the natural orientation of the ventricles and avoiding the major coronary arteries. During this process (about 3 h), the hearts were regularly massaged and moistened to maintain tissue elasticity.

##### Magnetic Actuation

The magnetic fields used to actuate the patches were generated by the MAS CardioMag (Multi‐Scale Robotic Lab, ETH Zurich) which is an eight‐coil electromagnetic navigation system (eMNS) capable of generating magnetic fields in a workspace of 20 cm × 20 cm × 20 cm. This eMNS can control the magnetic field direction and the magnetic field gradient to navigate magnetic catheters and magnetic microrobots. In the scope of this paper, only magnetic fields aligned with the *z*‐axis were used, so the eMNS was over dimensioned in terms of functionality and not representative of a dedicated MAS for MACPs. The heart containing the MACPs was placed on the system, as depicted in Figure S2 (Supporting Information). The two tubes were fixed with a clamp, allowing the heart to have some freedom of movement. After fixing the heart, both tubes were filled with water colored by rhodamine B, and the bubbles inside the heart were removed by tilting and squeezing the heart muscles. A flat glass Petri dish was placed under the heart to collect any drops from the heart due to small leakages. The heart motion and the water levels in both tubes were recorded simultaneously in all the experiments.

##### Data Acquisition and Analysis

To analyze the pumping performance, a digital camera (Canon EOS 5D Mark IV) was used to record the heart motion and the water levels during the experiments. After the experiments, the changing water levels were tracked with respect to time using an opensource software Tracker (Douglas Brown, 2019). The pumping volume was calculated by *V* = *πr* ^2^ (*h_t_* – *h*_0_), where *V* is the volumetric change, *r* is the inner radius of the tube, *h_t_* is the water level at time *t*, and *h*_0_ is the initial water level. The tracked data can be found in Figures S6–S10 (Supporting Information).

## Conflict of Interest

The authors declare no conflict of interest.

## Supporting information

Supporting Information

Supplemental Video 1
