# Supplementary material for: Magnetically Active Cardiac Patches as an Untethered, Non‐Blood Contacting Ventricular Assist Device
Source: Adv Sci (Weinh). 2020 Nov 27;8(1):2000726. doi: 10.1002/advs.202000726 (PMC7788498; doi:10.1002/advs.202000726)
Supplement: Supplementary file 1 — Supporting Information [file ADVS-8-2000726-s001.pdf]

© 2020 Wiley-VCH GmbH

ADVANCED SCIENCE
Open Access

Supporting Information

for *Adv. Sci.,* DOI: 10.1002/advs.202000726

Magnetically Active Cardiac Patches as an Untethered, Non-Blood Contacting Ventricular Assist Device

Hongri Gu, Thibaud Bertrand, Quentin Boehler, Christophe Chautems, Nikolay V. Vasilyev, and Bradley J. Nelson**

Copyright WILEY-VCH Verlag GmbH & Co. KGaA, 69469 Weinheim, Germany, 2020.

Supporting Information

Magnetically Active Cardiac Patches as an Untethered, Non-Blood Contacting Ventricular Assist Device

Hongri Gu, Thibaud Bertrand, Quentin Boehler, Christophe Chautems, Nikolay V. Vasilyev, Bradley J. Nelson**

Magnet configurations	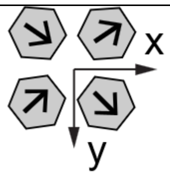		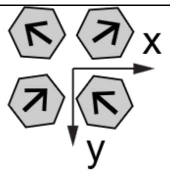		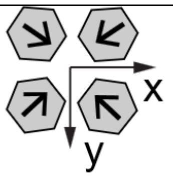		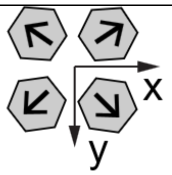	
	LV	RV	LV	RV	LV	RV	LV	RV
Relaxed Volume	21.4 mL	26.2 mL	21.4 mL	26.2 mL	21.4 mL	26.2 mL	21.4 mL	26.2 mL
Maximum Static Ejection Fraction (80 mT)	29.0%	42.3%	30.2%	44.3%	34.6%	58.3%	**36.6%**	**63.2%**
Maximum Dynamic Ejection Fraction (60 mT)	23.8%	39.3%	**24.8%**	29.0%	22.9%	35.9%	23.8%	**41.9%**

Table 1: Summary of maximum performances of the left ventricle (LV) and right ventricle (RV) for four different magnet configurations. The maximum ejection fraction is calculated by using the maximum volume change during the actuation divided by the relaxed volume. For static

performance, we used the experimental data from an 80 mT magnetic field in the z direction, as in **Fig. 2**. And a 60 mT oscillating magnetic field in the z direction at 0.25 Hz was used for dynamic performances, as in **Fig. 3**. The four configurations are shown with top view in this table, which are identical to the four configurations in **Fig 2** and **Fig. 3**. The black arrows represent the dipole directions of the hexagon magnets.

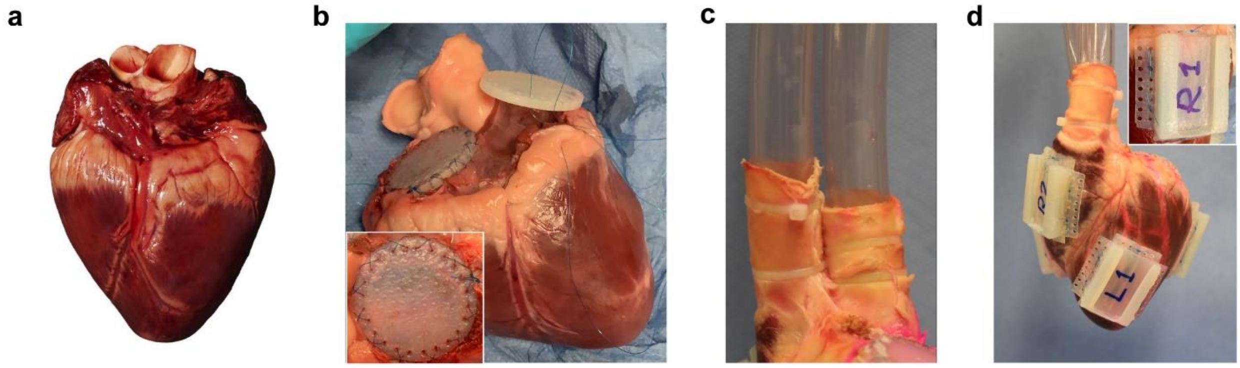

Figure S1: Preparation of the heart: (a) First, the pericardium was removed and then the excess part of the aorta and pulmonary artery. (b) Both the left and right atria were removed at the ventricular base. Both ventricles were cleaned with warm water and sutured with the sealing caps. After sutures, the gap between the tissue and the sealing cap was filled with cyanate glue. (c) Two PVC tubes were inserted into the aorta and pulmonary artery and fastened with zip ties and cyanate glue. (d) After the leakage check of the heart, the 3D-printed patches were sutured onto the ventricular walls. In total four patches were sutured onto the heart - two on the left ventricle and two on the right ventricle.

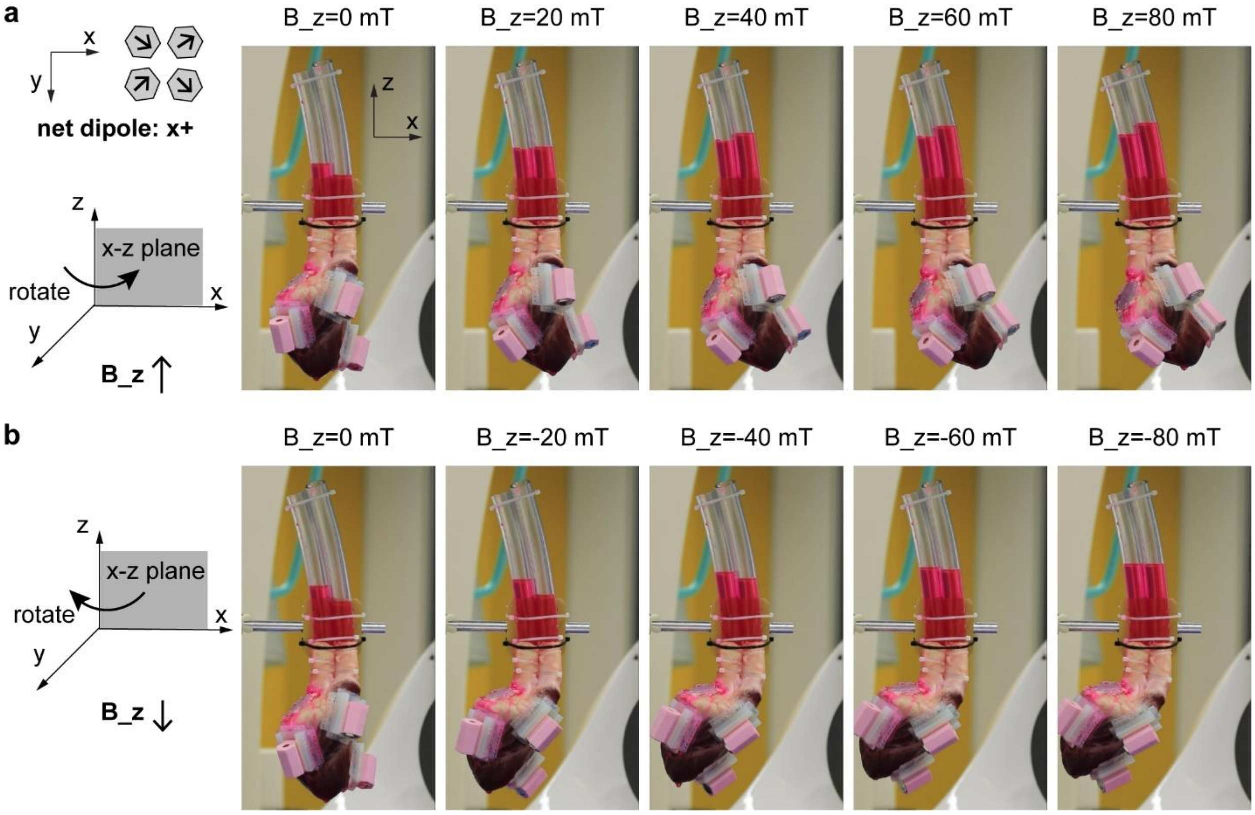

Figure S2: Static performances of the magnetically active cardiac patches with different magnet configurations (part one). In this configuration, there was a net dipole in the positive x direction. During the experiments, the heart rotated inside the x-z plane due to the net dipole attempting to align with the magnetic field. The motions of the heart can be seen in the above figure.

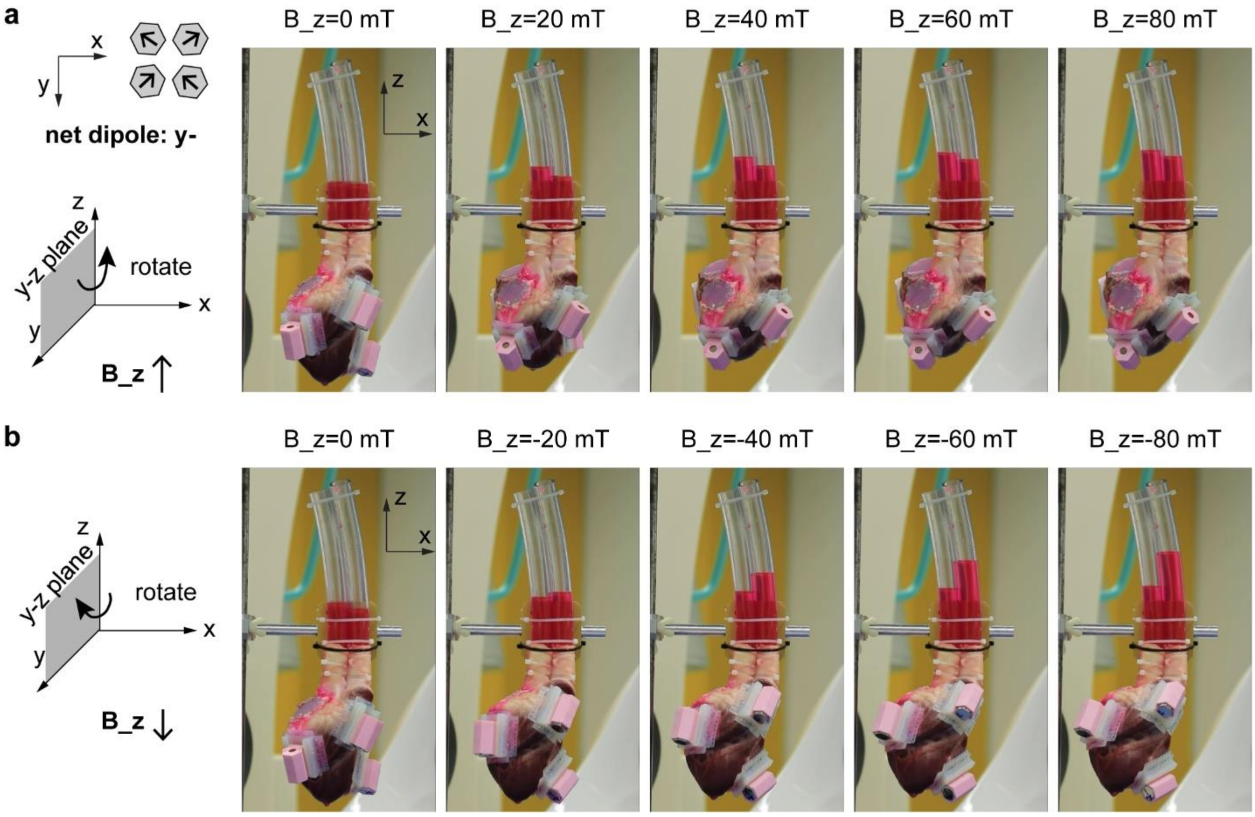

Figure S3: Static performances of the magnetically active cardiac patches with different magnet configurations (part two). In this configuration, there was a net dipole in the negative y direction. During the experiments, the heart rotated inside the y-z plane due to the net dipole attempting to align with the magnetic field. The motions of the heart can be seen in the above figure.

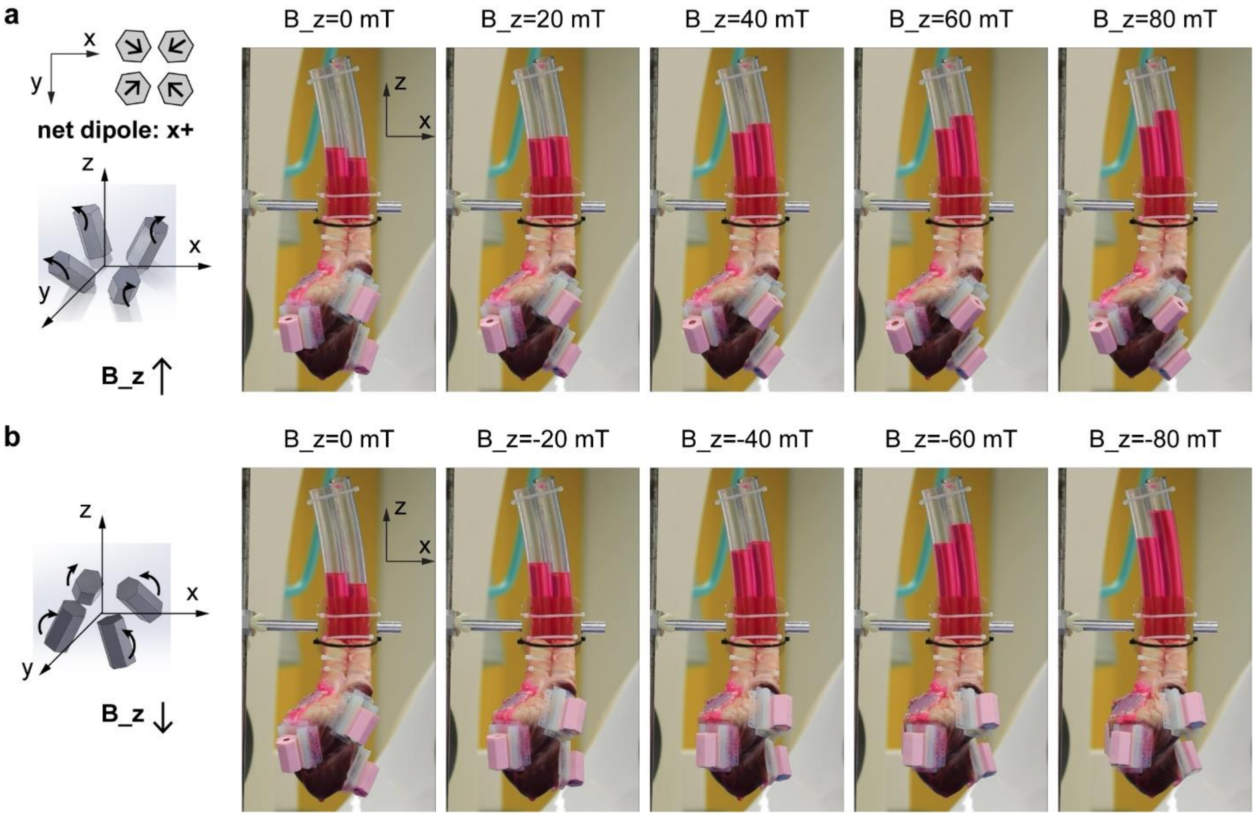

Figure S4: Static performances of the magnetically active cardiac patches with different magnet configurations (part three). In this configuration, there was no net dipole in the magnet arrangement. During the experiments, no obvious rotation was observed.

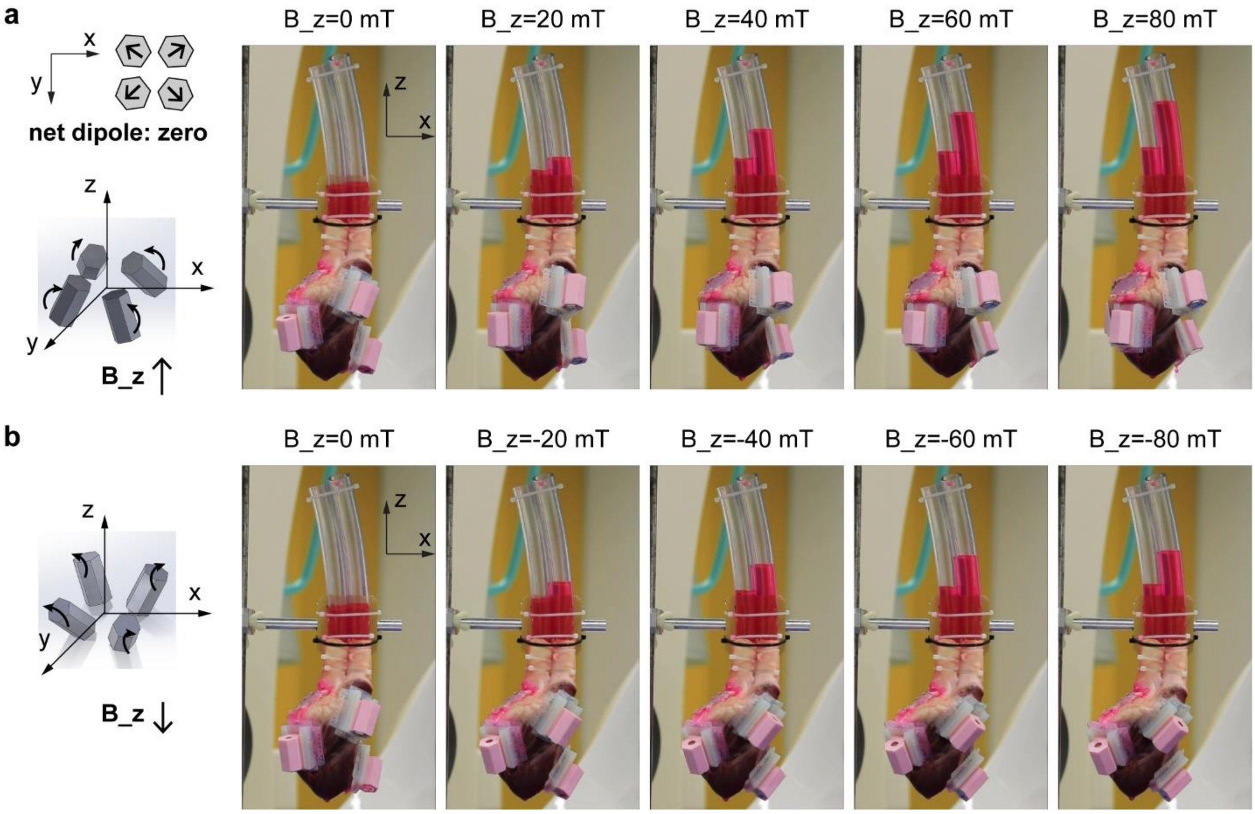

Figure S5: Static performances of the magnetically active cardiac patches with different magnet configurations (part four). In this configuration, there was no net dipole in the magnet arrangement. During the experiments, no obvious rotation was observed.

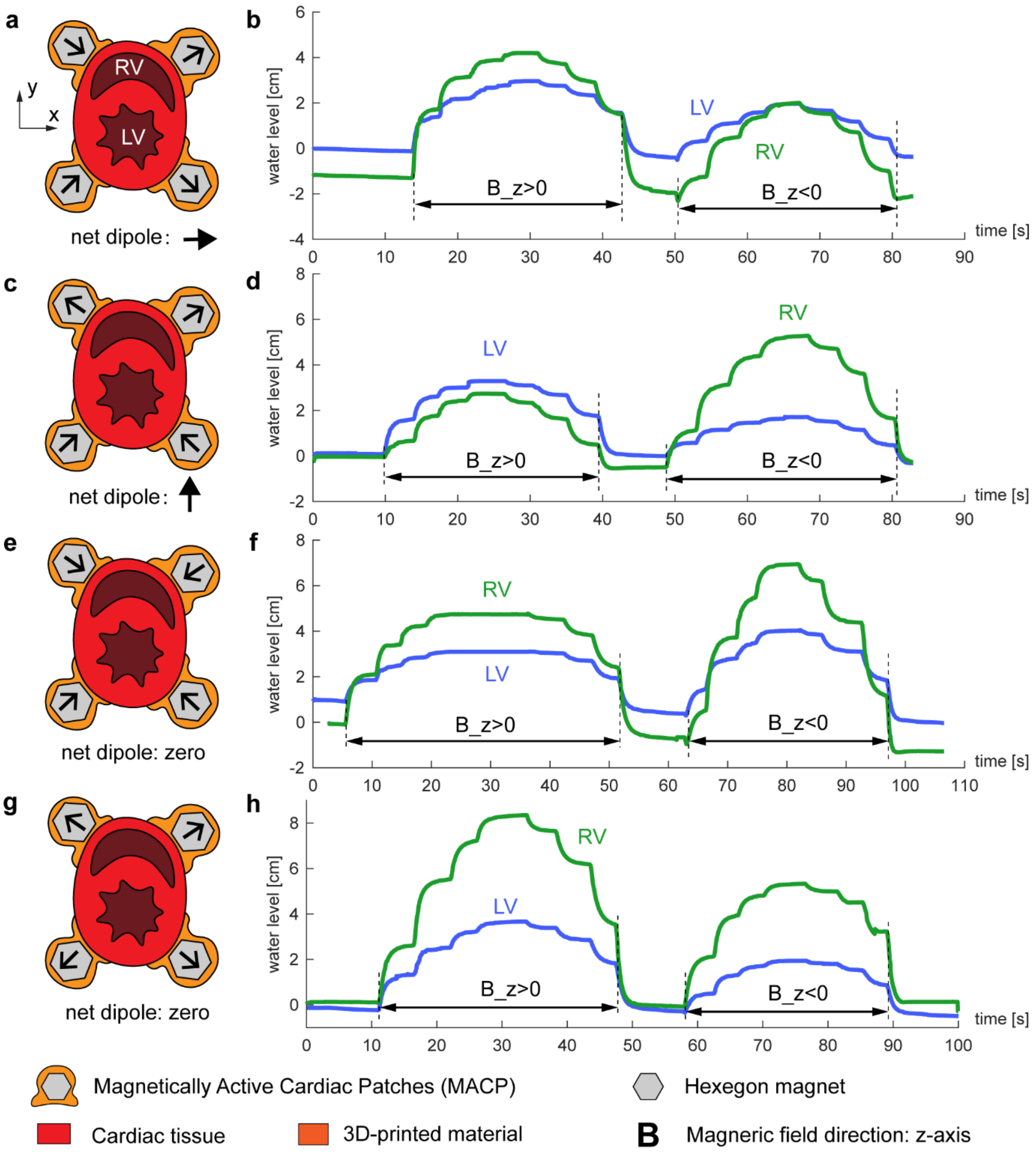

Figure S6: Step responses of the MCAPs with various magnet configurations. The magnetic field at z direction was increased and decreased with a constant 20 mT step. The maximum field of 80 mT was applied in both the positive and negative z directions. The results of the water levels were recorded and are shown in **Fig. b, d , f** and **h**. In each step we waited until the water level was stabilized. LV and RV stand for left ventricle and right ventricle, respectively.

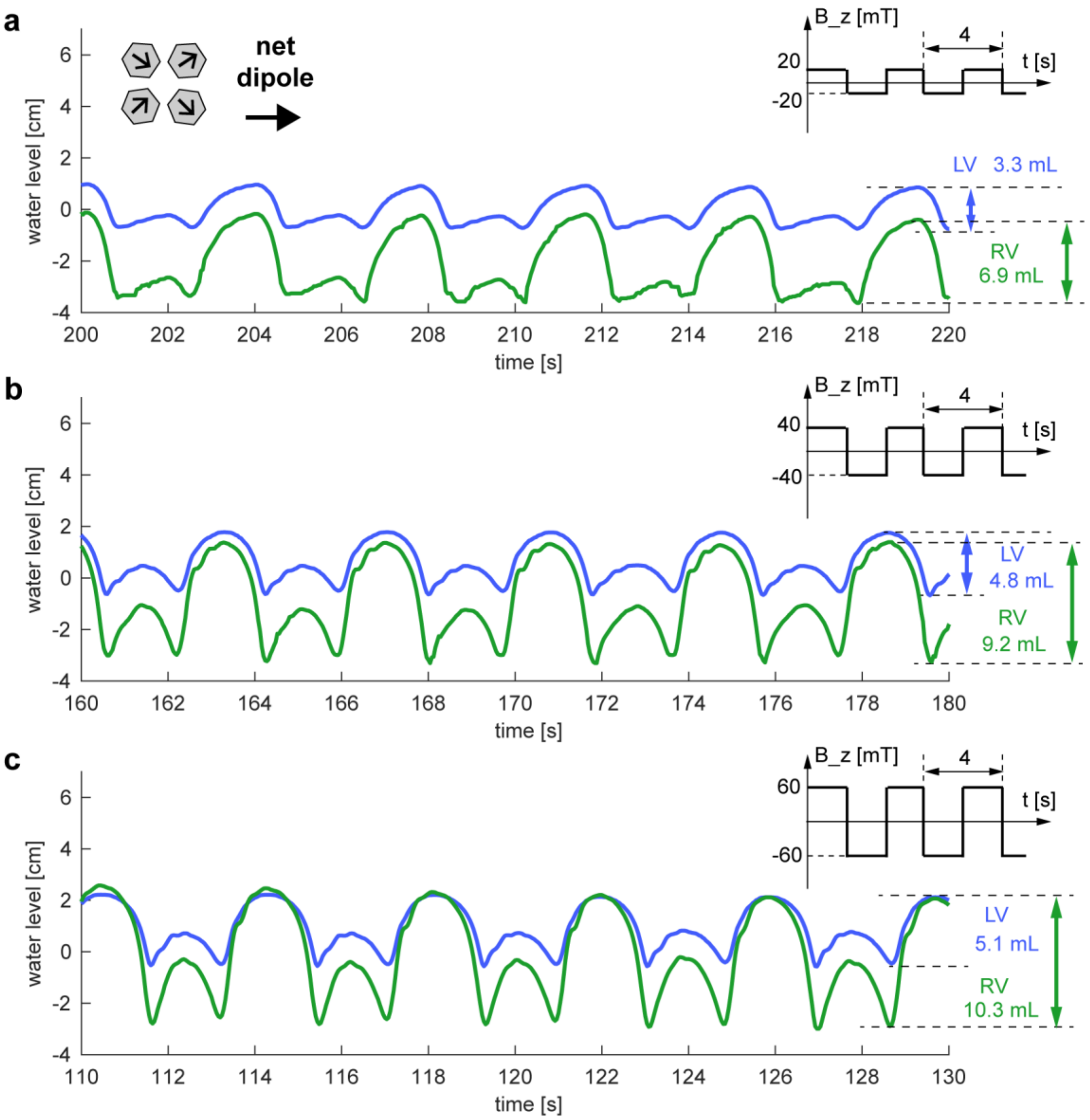

Figure S7: Dynamic performances of MACPs at 0.25 Hz with 20, 40 and 60 mT oscillating magnetic fields (part one). The magnet configuration is identical to **Fig. 2a**.

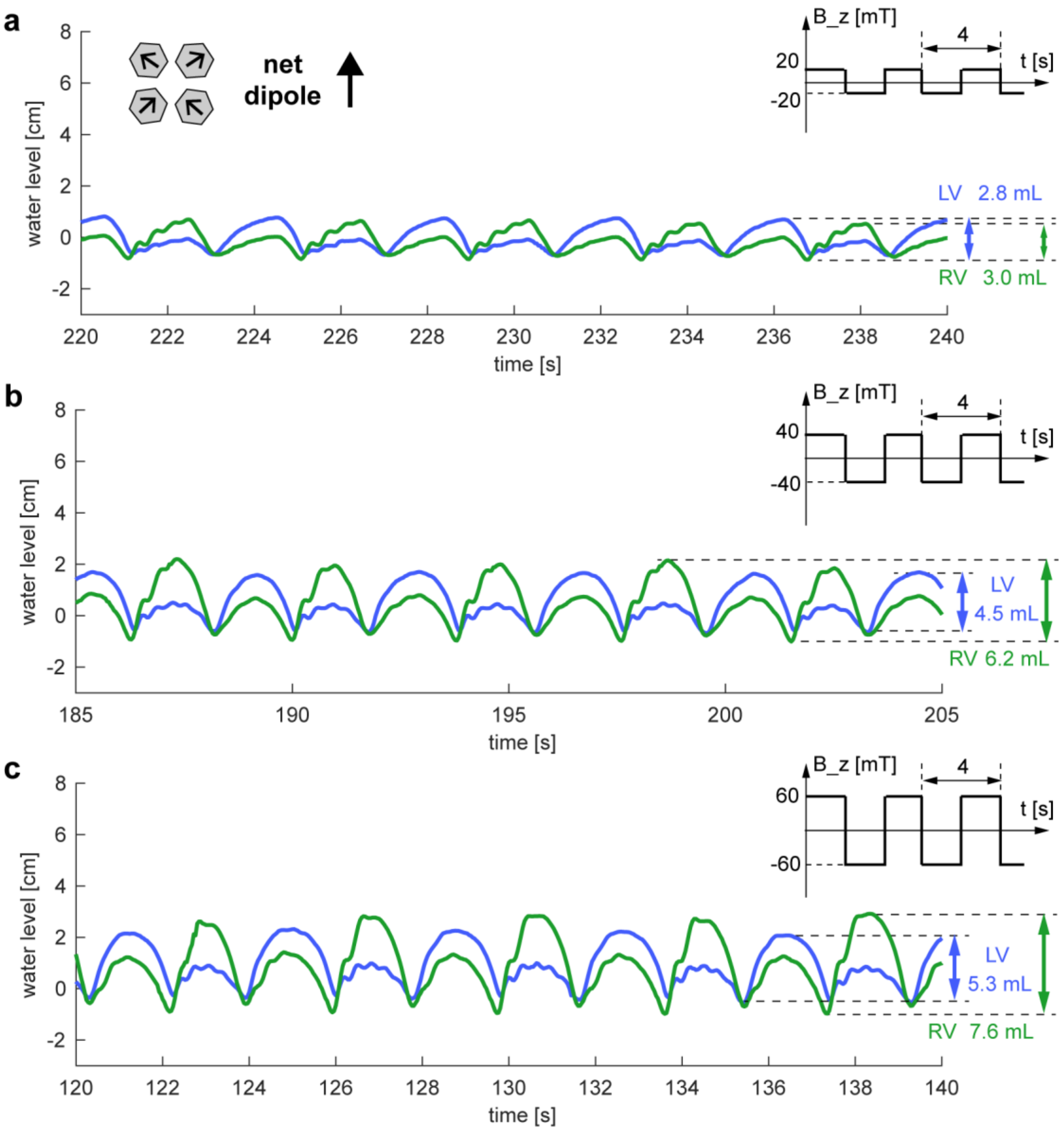

Figure S8: Dynamic performances of MACPs at 0.25 Hz with 20, 40 and 60 mT oscillating magnetic fields (part two). The magnet configuration is identical to **Fig. 2b**.

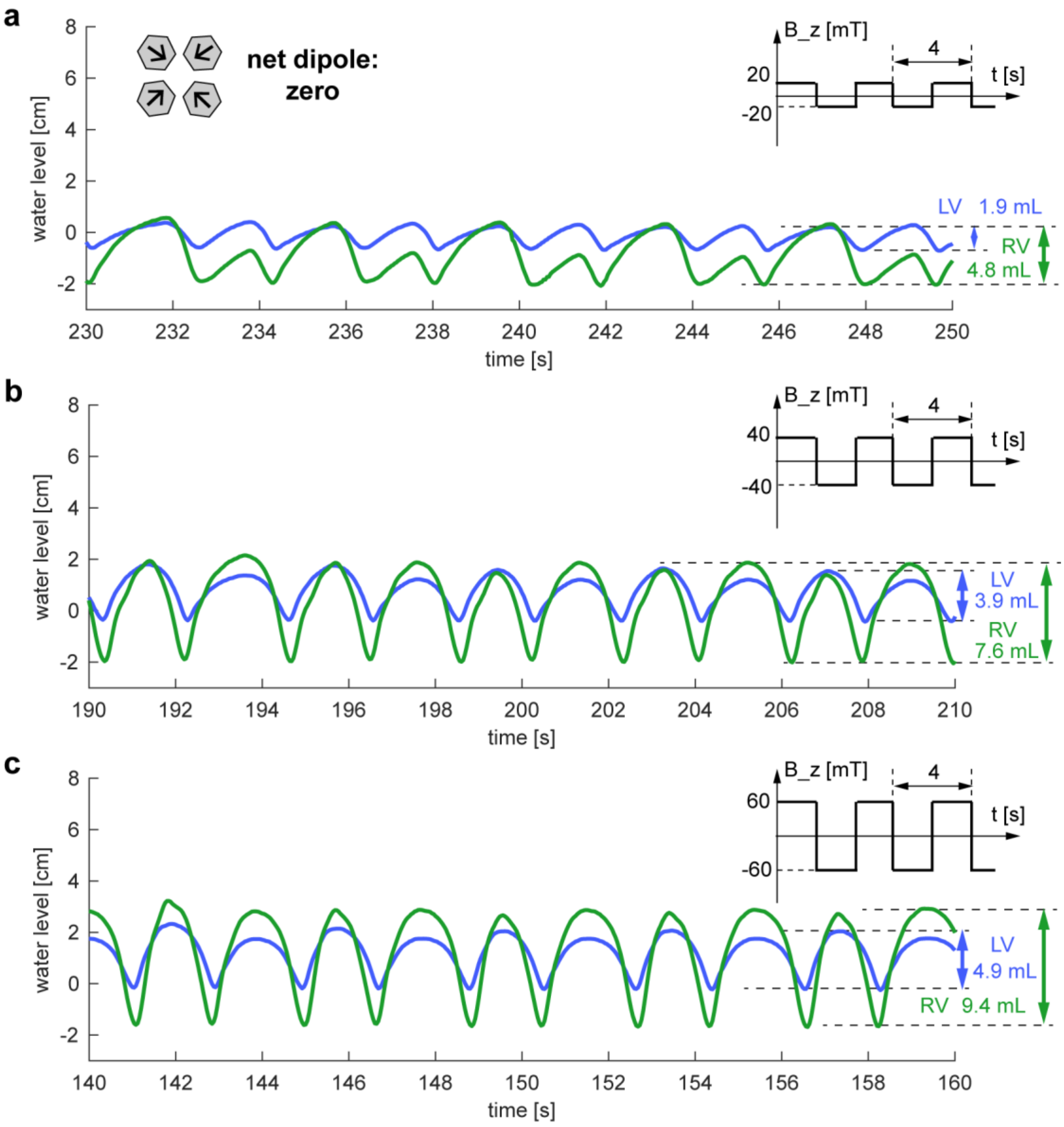

Figure S9: Dynamic performances of MACPs at 0.25 Hz with 20, 40 and 60 mT oscillating magnetic fields (part three). The magnet configuration is identical to **Fig. 2c**.

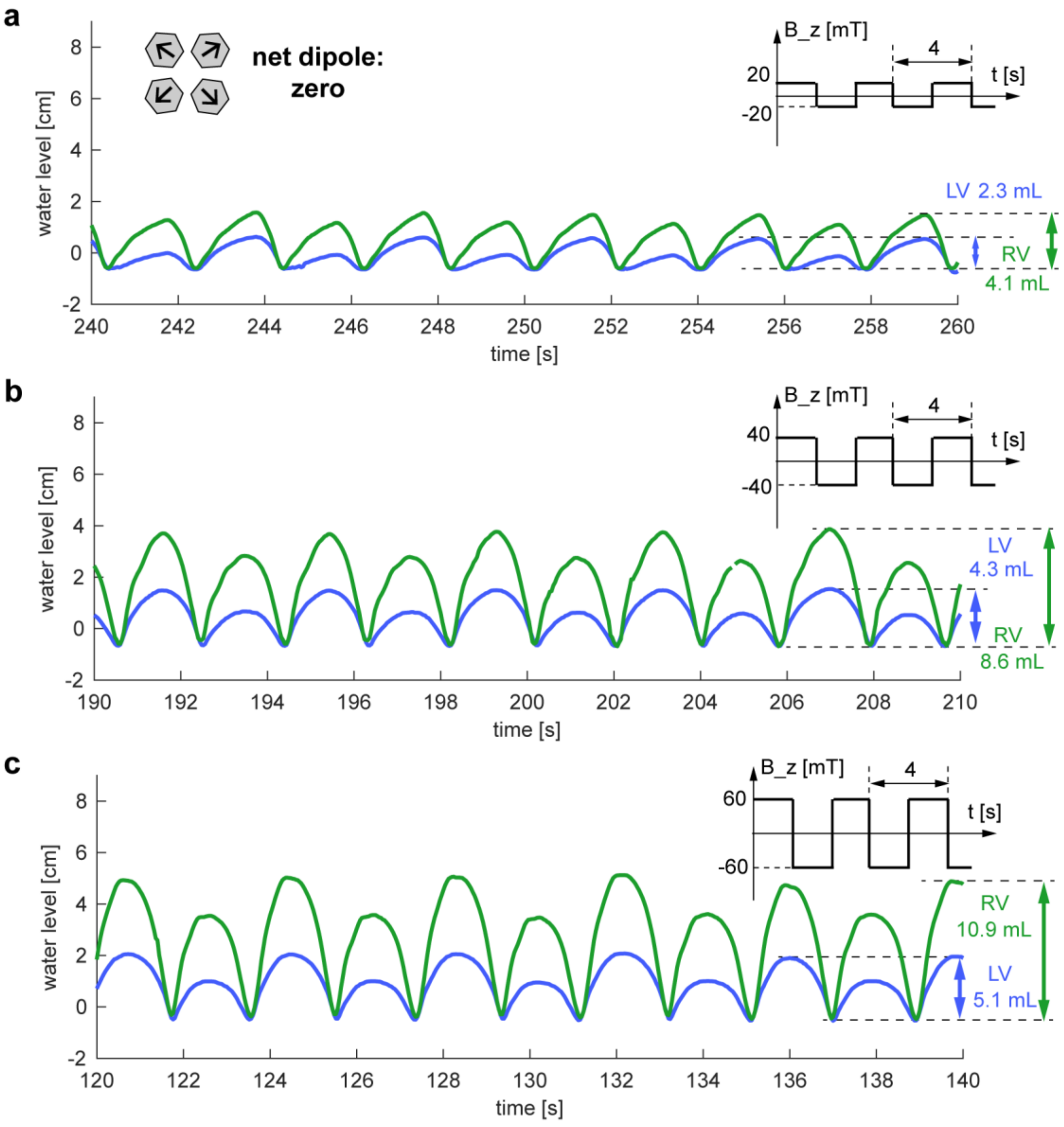

Figure S10: Dynamic performances of MACPs at 0.25 Hz with 20, 40 and 60 mT oscillating magnetic fields (part four). The magnet configuration is identical to **Fig. 2d**.

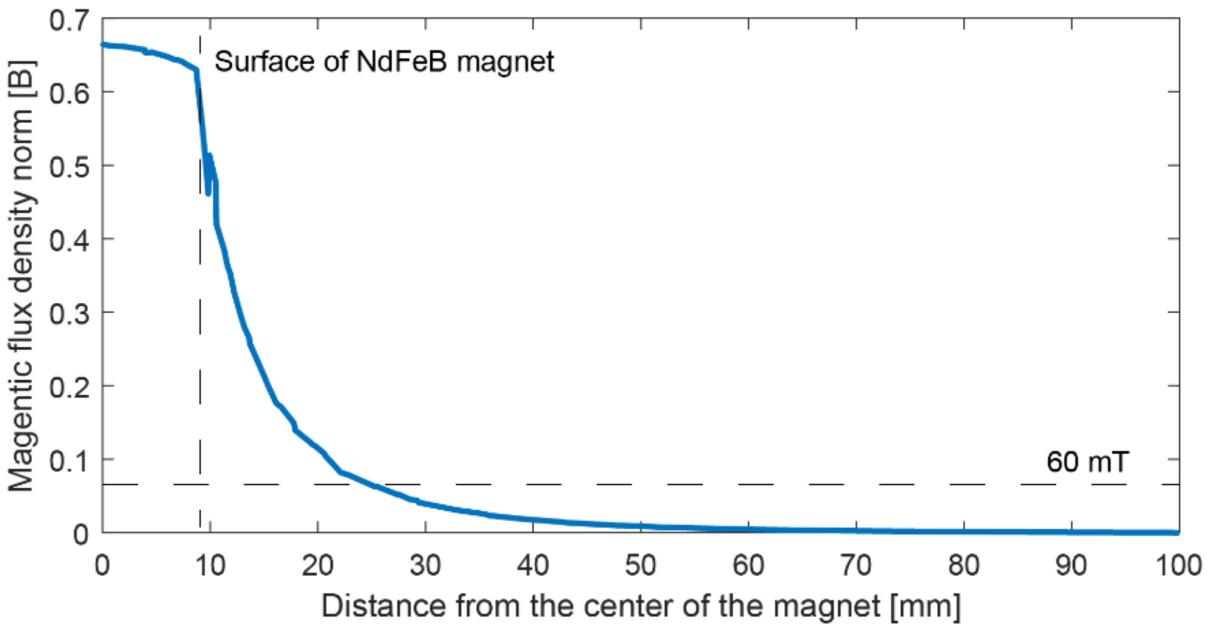

Figure S11. Simulation results of the magnetic field flux density around the single hexagon prism NdFeB magnet (length: 3 cm, hexagon length: 0.87 cm).

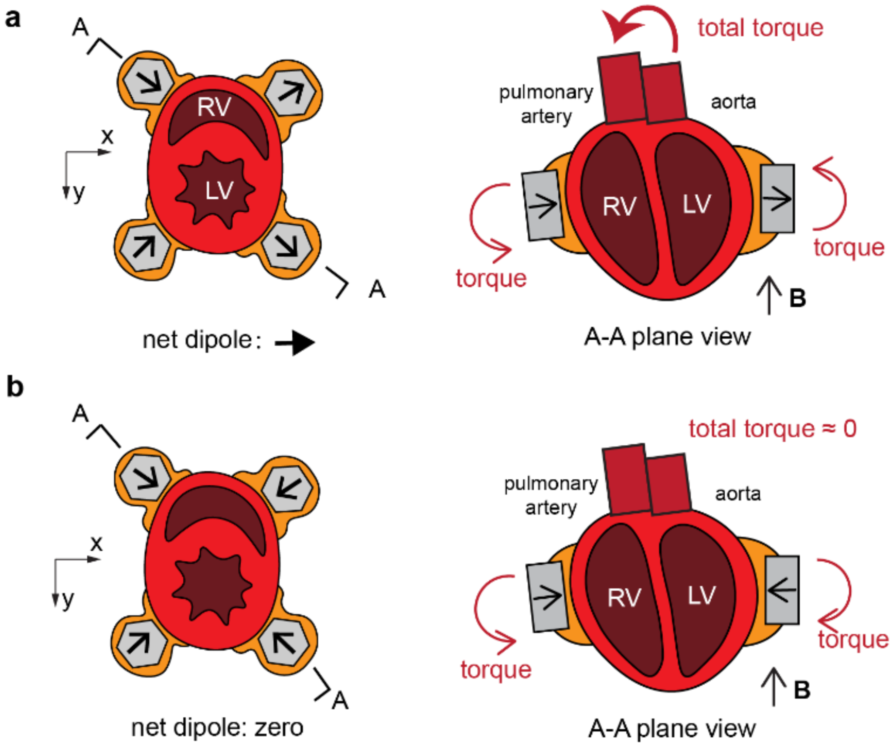

Figure S12. The comparison between the zero and non-zero net dipole arrangement. The non-zero net dipole arrangements create torque on the pulmonary artery and aorta, while the zero-dipole configuration have negligible total torque.
